# Comparative assessment of orthodontic clear aligner versus fixed appliance for anterior retraction: a finite element study

**DOI:** 10.1186/s12903-023-03704-6

**Published:** 2024-01-13

**Authors:** Qian Xia, Weixu Wang, Chunjuan Wang, Ge Feng, Chao Wang, Jinlin Song, Yubo Fan

**Affiliations:** 1 Stomatological Hospital of Chongqing Medical University, Chongqing, China; 2 Chongqing Key Laboratory of Oral Diseases and Biomedical Sciences, Chongqing, China; 3 Chongqing Municipal Key Laboratory of Oral Biomedical Engineering of Higher Education, Chongqing, China; 4 Key Laboratory of Biomechanics and Mechanobiology, Beijing Advanced Innovation Center for Biomedical Engineering, School of Biological Science and Medical Engineering, School of Engineering Medicine, State Key Laboratory of Virtual Reality Technology and Systems, Ministry of Education, Beihang University, No.37, Xueyuan Road, Beijing, 100083 China

**Keywords:** Clear aligner, Fixed appliance, Finite element analysis, Anterior retraction, Biomechanics

## Abstract

**Background:**

The aim of this study is to conduct a comparative evaluation of different designs of clear aligners and examine the disparities between clear aligners and fixed appliances.

**Methods:**

3D digital models were created, consisting of a maxillary dentition without first premolars, maxilla, periodontal ligaments, attachments, micro-implant, 3D printed lingual retractor, brackets, archwire and clear aligner. The study involved the creation of five design models for clear aligner maxillary anterior internal retraction and one design model for fixed appliance maxillary anterior internal retraction, which were subsequently subjected to finite element analysis. These design models included: (1) Model C0 Control, (2) Model C1 Posterior Micro-implant, (3) Model C2 Anterior Micro-implant, (4) Model C3 Palatal Plate, (5) Model C4 Lingual Retractor, and (6) Model F0 Fixed Appliance.

**Results:**

In the clear aligner models, a consistent pattern of tooth movement was observed. Notably, among all tested models, the modified clear aligner Model C3 exhibited the smallest differences in sagittal displacement of the crown-root of the central incisor, vertical displacement of the central incisor, sagittal displacement of the second premolar and second molar, as well as vertical displacement of posterior teeth. However, distinct variations in tooth movement trends were observed between the clear aligner models and the fixed appliance model. Furthermore, compared to the fixed appliance model, significant increases in tooth displacement were achieved with the use of clear aligner models.

**Conclusions:**

In the clear aligner models, the movement trend of the teeth remained consistent, but there were variations in the amount of tooth displacement. Overall, the Model C3 exhibited better torque control and provided greater protection for posterior anchorage teeth compared to the other four clear aligner models. On the other hand, the fixed appliance model provides superior anterior torque control and better protection of the posterior anchorage teeth compared to clear aligner models. The clear aligner approach and the fixed appliance approach still exhibit a disparity; nevertheless, this study offers a developmental direction and establishes a theoretical foundation for future non-invasive, aesthetically pleasing, comfortable, and efficient modalities of clear aligner treatment.

**Supplementary Information:**

The online version contains supplementary material available at 10.1186/s12903-023-03704-6.

## Introduction

Prominent facial deformity, a prevalent malocclusion in orthodontic clinical practice, significantly impacts facial aesthetics. To enhance the lateral appearance in cases of dental or mild bony protrusions, optimal results can be achieved by extracting the first premolar and utilizing a fixed appliance or clear aligner for maximizing internal retraction of the anterior teeth. Fixed orthodontic maxillary micro-implant anchorage structures provide effective and safe treatment for cases of protrusion [1]. In contrast, achieving precise control over the three-dimensional movement of teeth using clear aligners necessitates a combination of mini-screws, power ridges, overtreatment, or power arms to optimize anterior torque control and ensure posterior anchorage during anterior retraction [2–9]. However, both clear aligners and fixed orthotics currently possess several limitations including potential trauma associated with micro-implant, aesthetic concerns, possible increase in unnecessary reciprocating motion, and treatment uncertainty [2, 7, 10–13].

To enhance the aesthetic appeal, minimize invasiveness, and optimize efficiency in retracting anterior teeth during clear aligner therapy, we have developed two novel design models for clear aligner retraction. The first modification involves a palatal plate-shaped clear aligner, which can now be directly printed using 3D-printing technology. This advancement improves the design parameters of aligners, including configuration, strength, elasticity, and thickness [14–17], thereby enhancing their therapeutic efficacy. The second one is a Lingual Retractor that utilizes advanced 3D-printing technology to create a compound structure specifically designed for seamless integration with clear aligners. Recently, our research group has developed patient-specific attachments utilizing 3D printing technology that have been validated through finite element analysis to exhibit superior anterior tooth anchorage in comparison to alternative attachments during maxillary molar distalization [18]. Several studies have documented that successful treatment of patients requiring anterior retraction can be achieved by combining a Double J retractor with a fixed appliance [19, 20]. Additionally, the bracket re-bonding procedure, which is a complex operation, may also be necessary. Moreover, the utilization of a palatal micro-implant remains indispensable. The incorporation of clear aligners in conjunction with tongue retractors is expected to enhance the convenience and efficacy of anterior tooth retraction.

Orthodontic clear aligners can be fabricated from either traditional thermoplastic materials or light-cured shape memory resins. The development of innovative materials has played a pivotal role in enhancing the effectiveness of clear aligners. Currently, there is an abundance of research available on clear aligner materials, with more comprehensive investigations accessible in scholarly articles authored by Ning and Naohisa [21–24]. It is worth noting that the meticulous design of clear aligner morphology and its composite force system structure holds paramount importance. For instance, in the case of anterior internal retraction, a power ridge was incorporated into the clear aligner design to effectively control maxillary anterior teeth torque [25]. However, it has been observed that the utilization of a power ridge frequently results in dislocation of clear aligners, subsequently exerting an impact on orthodontic outcomes [26]. Additionally, micro-implant anchorage composite force systems have been explored for anterior teeth retraction; however, many patients are reluctant to undergo this invasive treatment modality [7]. Despite these challenges, there remains a lack of effective noninvasive and aesthetic anterior teeth retraction using clear aligners. Recently, we employed simulation methodology to investigate the biomechanical characteristics and retraction effects of our innovative designs for two non-invasive and aesthetically pleasing models using clear aligners. Nonetheless, comprehensive comparative and biomechanical analyses regarding the clinical efficacy of anterior teeth retractions versus fixed appliances are still insufficient.

Therefore, the purpose of this study was to compare and evaluate the differences among various design of clear aligners, as well as to assess the disparities between the clear aligner model and the fixed appliance. The study encompasses five distinct clear aligner retraction models and one fixed appliance retraction model (Model C0 Control, Model C1 Posterior Micro-implant, Model C2 Anterior Micro-implant, Model C3 Palatal Plate and Model C4 Lingual Retractor, and Model F0 Fixed Appliance). In this study, employing numerical modeling, we conducted an analysis and comparison of the therapeutic efficacy of various orthodontic appliances as well as the biomechanical response of dental and periodontal ligament structures in orthodontics.

## Materials and methods

### Acquisition of medical image data

A patient with permanent dentition and maxillary bone protrusion requiring extraction of the first premolar was selected from the Department of Orthodontics at Affiliated Stomatological Hospital of Chongqing Medical University. The present study was granted ethical approval by the Stomatological Hospital of Chongqing Medical University (2023) 056. Cone-beam computed tomography (CBCT) with specific parameters (120 kVp; 5 mA; voxel size of 0.4 mm; Kava, Biberach, Germany) and 3D intraoral scanning were employed to acquire DICOM (Digital Imaging and Communications in Medicine) data.

Inclusion criteria for the study were as follows: (a) Complete development of the jaw and presence of all teeth, excluding third molars; (b) Adult patients with maxillary protrusion, ANB＞4°, U1-SN < 105°, and extraction of the maxillary first premolar for orthodontic treatment [27]; (c) Healthy dentition without extensive fillings, no history of root canal treatment, and absence of restoration crowns or dental implants; (d) Periodontal and temporomandibular joints exhibited normal conditions; (e) Complete cone-beam computed tomography (CBCT) and intraoral scan data were available.

Exclusion criteria: (a) The clinical crown height on the palatal side of the maxillary posterior teeth is insufficient, measuring less than 4 mm; (b) The root length of the maxillary posterior teeth is inadequate, with a root to crown ratio (R/C) ≤ 1 [28]; (c) Patients with a history of maxillary surgery, trauma, or tumor are included; (d) Developmental deformities affecting the integrity and structure of the jaw, such as severe asymmetry and cleft palate in the maxilla.

### The construction of orthodontic model

**Fig. 1:**
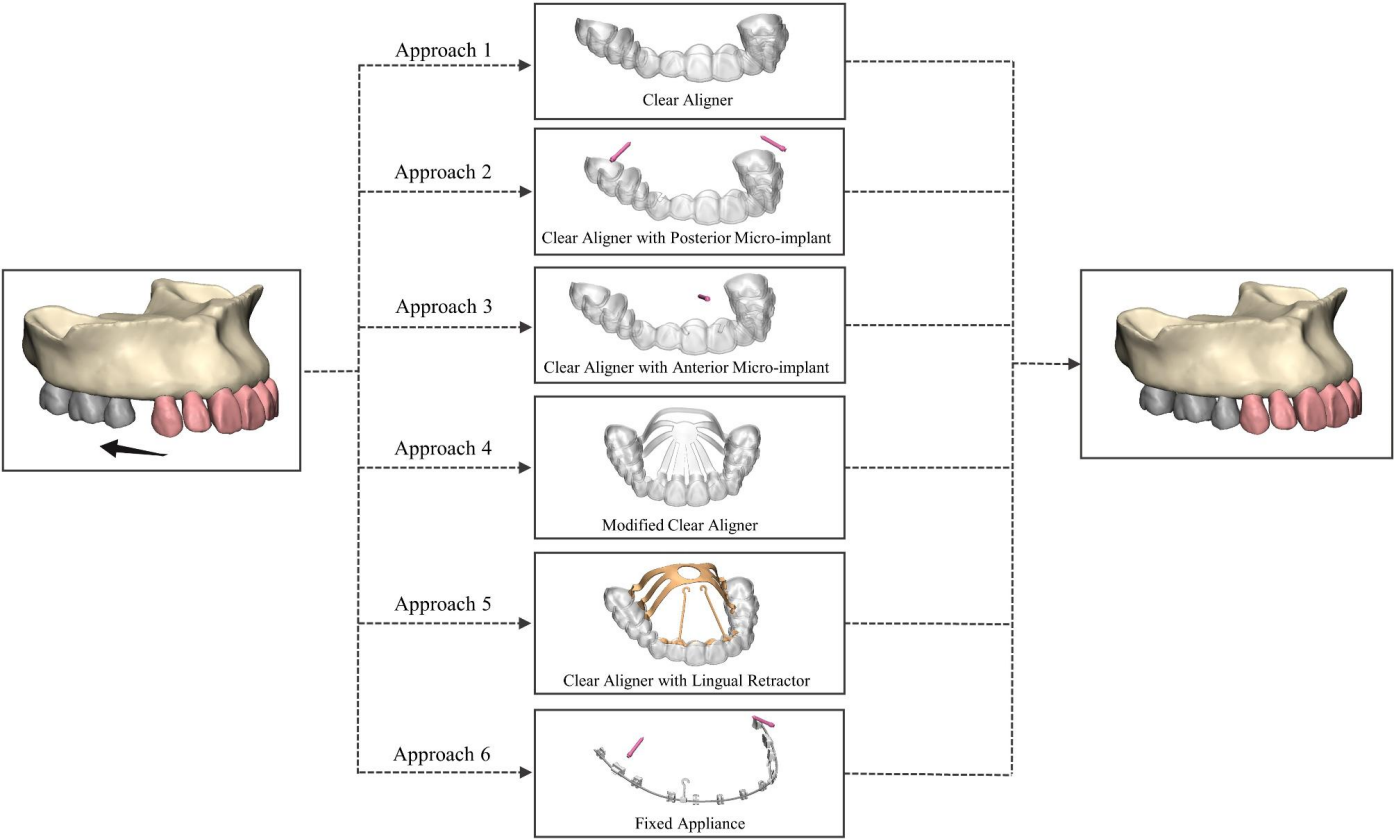
3D finite element model design of anterior teeth retraction approach

The DICOM data was imported into the Mimics system (Materialize, Belgium). The threshold range was adjusted based on grayscale differences to segment preliminary 3D models of the maxilla and dentition. Geomagic Studio software (Geomagic, USA) was used for surface fine-tuning and smoothing, followed by generating CAD models through autosurfacing. By utilizing the Boolean operation and offset functions in 3-matic software, we established a PDL with an average thickness of 0.2 mm and cortical bone of 2.0 mm, considering cancellous bone as residual material. The extraction dentition model was created by removing the first premolars and their PDL. We obtained a model of anterior tooth retraction of 0.2 mm using six retraction approaches (five clear aligner approaches and one fixed appliance approach), as shown in Fig. 1 [29]. The clear aligner was developed by applying an external offset on the post-retraction model with a thickness of 0.75 mm [30].

**Fig. 2:**
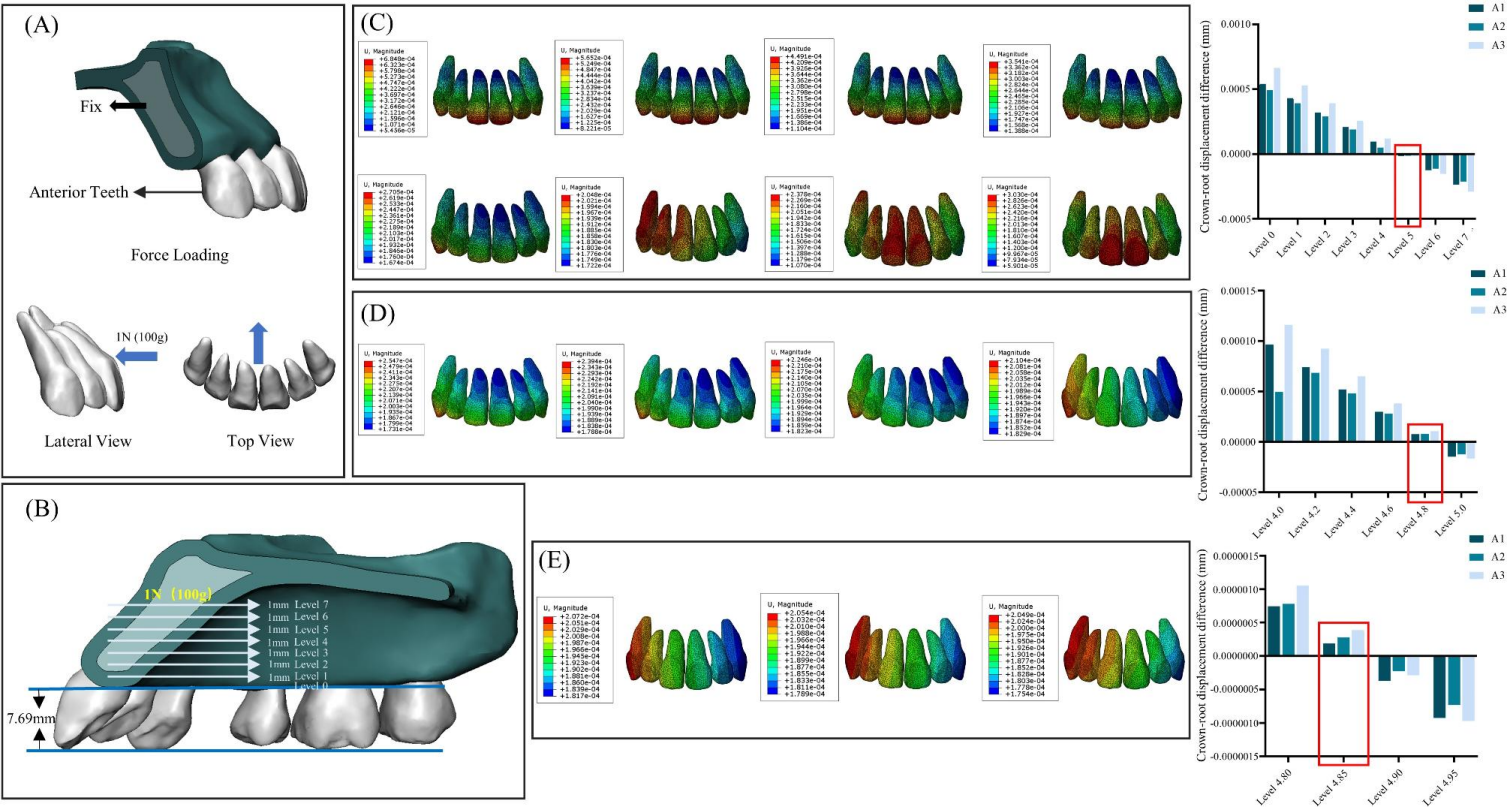
Design models for determining the center of resistance: (**A**) Boundary condition and force loading of retraction units; (**B**) The approximate force level axis; (**C**), Comparison of the maximum initial displacements (blue to red reflects lower to higher displacement) of the teeth (level 0–7); (**D**) Comparison of the maximum initial displacements of the teeth (level 4.0–5.0); (**E**), Comparison of the maximum initial displacements of the teeth (level 4.80–4.95)

**Fig. 3:**
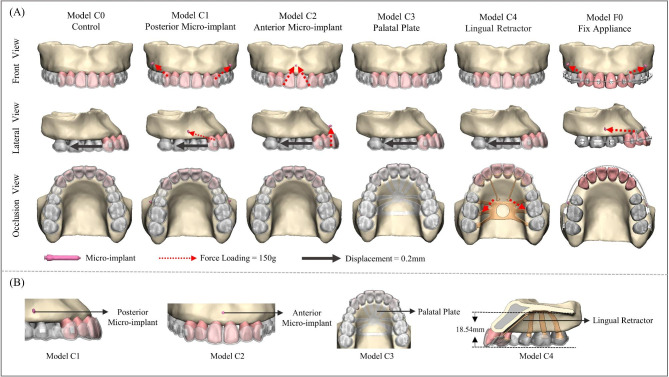
(**A**) Grouping about retraction treatment: Model C0 Control, Model C1 Posterior MI, Model C2 Anterior MI, Model C3 Palatal Plate, Model C4 Lingual Retractor, Model F0 Fix Appliance. Red arrow represents the applied force loading (150 g) from precision cutting or hook to Micro-implants. Black arrow represents the same activation (0.2 mm retraction) of anterior teeth. (**B**) Details of Model C1, Model C2, Model C3 and Model C4, the distance from the traction point to the occlusion plane is 18.54 mm

One of the clear aligner retraction models combined a clear aligner with a 3D printed lingual retraction hook and a 3D printed palatal plate. In this simulation, the anterior teeth were considered as a retraction unit. The lingual retractor and palatal plate were bonded to the tooth surface through the base plate [31]. The thickness of both the lingual retractor and the palatal plate was 0.5 mm (Supplementary Fig. 1). The center of resistance (CR) is considered the fundamental reference point for controlled tooth movement, and the height of the lingual retraction hook was determined based on the center of resistance (CR) of the retraction unit. The retraction unit models were assigned the property of rigidity. The mesial-distal truncated surfaces of the maxilla were firmly constrained (Fig. 2, A). In order to ascertain the vertical position of the center of resistance (CR) for the retraction unit, a 100 g horizontal force was exerted in close proximity to the median sagittal plane and parallel to the occlusal plane, inducing lingual retraction (Fig. 2, A). In addition, the point of force application (level 0) was precisely positioned on the alveolar ridge roof of the posterior teeth, at a distance of 7.69 mm from the incisal edge (Fig. 2, B). Commencing from level 0, it was incrementally advanced towards the root in perpendicular alignment with the occlusal plane at intervals of 1 mm up to level 7, which corresponded closely to the apex of the anterior teeth. during anterior retraction. All components were imported into finite element (FE) software for calculations. The difference between the displacement of the root tip and crown displacement was defined as the crown-root differential displacement. The center of resistance (CR) level is defined as the point where the differential displacements of anchorage units are close to 0. After step-by-step subdivision of the loading calculation, we determined that the vertical position of the center of resistance (CR) is at 4.85 mm. Our clear aligner force system consists of a lingual retraction hook and a clear aligner, which shifts the center of resistance (CR) position towards the root due to force exerted on the crown section. we selected a position 6 mm above the CR as the length for lingual retraction hook (i.e., 18.54 mm above occlusal plane) (Supplementary file 1, Supplementary Fig. 2), which was close to the hard palate. (Fig. 3, B). A posterior traction site was designed using a 3D printed device, uniting six posterior teeth for anchorage (Fig. 3, A). Additionally, the traction points can be customized based on clinical needs.

The construction of five types of clear aligner retraction models (including the Model C0 Control, Model C1 Posterior Micro-implant, Model C2 Anterior Micro-implant, Model C3 Palatal Plate and Model C4 Lingual Retractor) and one Fixed retraction model (Model F0 Fix Appliance) is illustrated in Fig. 3. The Model C0 served as the control group for the clear aligner model, consisting solely of clear aligners. In Model C1, a micro-implant was positioned between the second premolar and first molar, 5 mm above the alveolar ridge’s highest point, at an angle of 60° to the maxillary occlusal plane, with an intraosseous length measuring 8 mm. The force of 150 g was applied [32, 33]. In Model C2, a micro-implant was positioned between the central incisors to apply the force of 150 g by directing it towards the lingual side through the precision cut [7]. Model C3 incorporated a palatal lateral plate that seamlessly integrated with the clear aligner in terms of thickness and material, which could be obtained through cutting. Additionally, Model C4 combined a clear aligner with a 3D printed lingual retraction device. Furthermore, the 3D printed device was generated using Mimics software. The lingual retraction hook was positioned 6 mm above the center of resistance (CR) (18.54 mm above the occlusal plane) and was designed and modeled using computer-aided design software SolidWorks (Dassault, France) (Fig. 3, A). Buccal surfaces of the canine featured vertical rectangular attachments measuring 3*2*1mm, while horizontal rectangular attachments of the same dimensions were designed on the buccal surfaces of both second premolar and first molar in all clear aligner models. Model F0, a commonly used clinical retraction system, comprised a relatively rigid rectangular archwire (0.018 × 0.025inch), a posterior micro-implant, and an anterior retraction hook with a height of 7 mm [34]. Additionally, a retraction force of 150 g was applied [35, 36].

### Material properties and meshing

The models were assembled and imported into ABAQUS software (SIMULIA, France). Each study subject was assumed to possess continuous homogeneity, isotropy, and a linear elastic material constitutive model. The material properties of the components, obtained from previous studies, are summarized in Table 1 [29, 37–45]. The meshing of the three-dimensional models was performed using the C3D10M element type, also known as a modified tetrahedral quadratic element that is particularly suitable for contact calculations. The number of nodes and mesh is approximately presented in Table 1.

**Table 1:** Material properties and element number

Component	Young’s modulus (MPa)	Poisson’s ratio	Nodes	Elements
Teeth	18,600	0.31	232,722	130,616
PDL	0.68	0.48	125,028	63,489
Cortical bone	13,700	0.3	217,757	120,647
Cancellous bone	1370	0.3	111,373	60,362
Clear aligner	816.31	0.3	123,155–162,229	64,300–67,937
Attachment	12,500	0.36	5850	2683
Power arm	200,000	0.3	5265	2537
3D printed attachment	235,000	0.33	52,082	24,438
Archwire	200,000	0.3	24,109	10,536
Bracket	210,000	0.3	30,317	14,254
Micro-implant	114,000	0.34	4871	2709

### Boundary constraints and contact conditions

The base of the maxilla was constrained to prevent any rotation or displacement from occurring. The contact relationships between the cortical and cancellous bone, alveolar bone and periodontal ligament (PDL), teeth and PDL, attachment and corresponding teeth, micro-implant and jaws, 3D printed lingual retractor and corresponding teeth, archwire and anterior teeth, as well as power arm and archwire were defined as bonded connections. The outer surface of the crown and the inner surface of the clear well as the attachment’s outer surface and the clear aligner’s inner surface, are considered non-linear face-to-face contacts. The tangential direction between these two contact surfaces is set to frictional with a coefficient of 0.2 [38, 46]. The coefficient of friction between bracket slots and archwire is assumed to be 0.2 [47–49]. The y-axis of the global coordinate system represents the vertical direction, with positive values defined as perpendicular to the occlusal plane towards the root. The local coordinate system is established for each tooth due to variations in mesiodistal and buccolingual directions. The x-axis represents the mesiodistal direction, where the x-value is defined as the distal direction and positive values are assigned to this direction. The z-axis represents the buccopalatal direction, with positive values defined for the palatal direction. Reference points were selected at the incisal midpoint and root apex of the incisors, cusp tip and root apex of the canines, buccal cusp tip and lingual cusp tip of second premolar, mesial buccal cusp tip, distal buccal cusp tip, mesial lingual cusp tip and distal lingual cusp tip of first molar, and mesial buccal cusp tip, distal buccal cusp tip and lingual cusp tip of second molar.

### Calculation and analysis

Due to the bilateral symmetry of the model employed in this study, we specifically selected the right maxillary tooth and periodontal ligament (PDL) for meticulous analysis. Nonlinear iterative calculations were conducted using ABAQUS software (SIMULIA, France), yielding comprehensive results encompassing displacement of teeth and aligners, as well as von-Mises equivalent stress experienced by both PDL and aligners.

## Results

### Determining the center of resistance

The displacement distribution and crown-root displacement differences of the six anterior teeth were illustrated in Fig. 2. As the center of resistance (CR) vertical position approached, the sagittal crown-root displacement difference tended to approach zero. Specifically, at level 4, the central incisor, lateral incisor, and canine exhibited positive crown-root displacement differences of 9.65E-05 mm, 4.96E-05 mm, and 1.20E-05 mm respectively. However, at level 5, these values became negative with respective crown-root displacement differences of -1.49E-05 mm for the central incisor, -1.24E-05 mm for the lateral incisor, and − 1.66E-05 mm for the canine. The force level axis from level 4.0 to 5.0 was meticulously sectioned at intervals of 0.2 mm for the various points of force application, as depicted in Fig. 2, D. At level 4.8, the crown-root displacement differences of the central incisor, lateral incisor, and canine were positive: 7.43E-06 mm, 7.79E-06 mm, and 1.07E-05 mm respectively. At level 5.0, the crown-root displacement differences of these teeth were negative with values consistent with those previously described. Subsequently, the force level axis from level 4.8 to 5 was meticulously sectioned every increment of 0.05 mm for the different points of force application shown in Fig. 2, E. At level 4.85 (Fig. 2, E), the difference in crown-root displacement between, lateral incisor and canine approached zero; specifically measuring at approximately: 1.86E-06 mm (central incisor), 2.75E-06 mm (lateral incisor) and 3.89E-06 mm (canine). Therefore, we considered this position as representing the vertical height of the center of resistance (CR).

### Comparison of the maximum displacements of the central incisor, lateral incisor, and canine in sagittal dimension

**Fig. 4:**
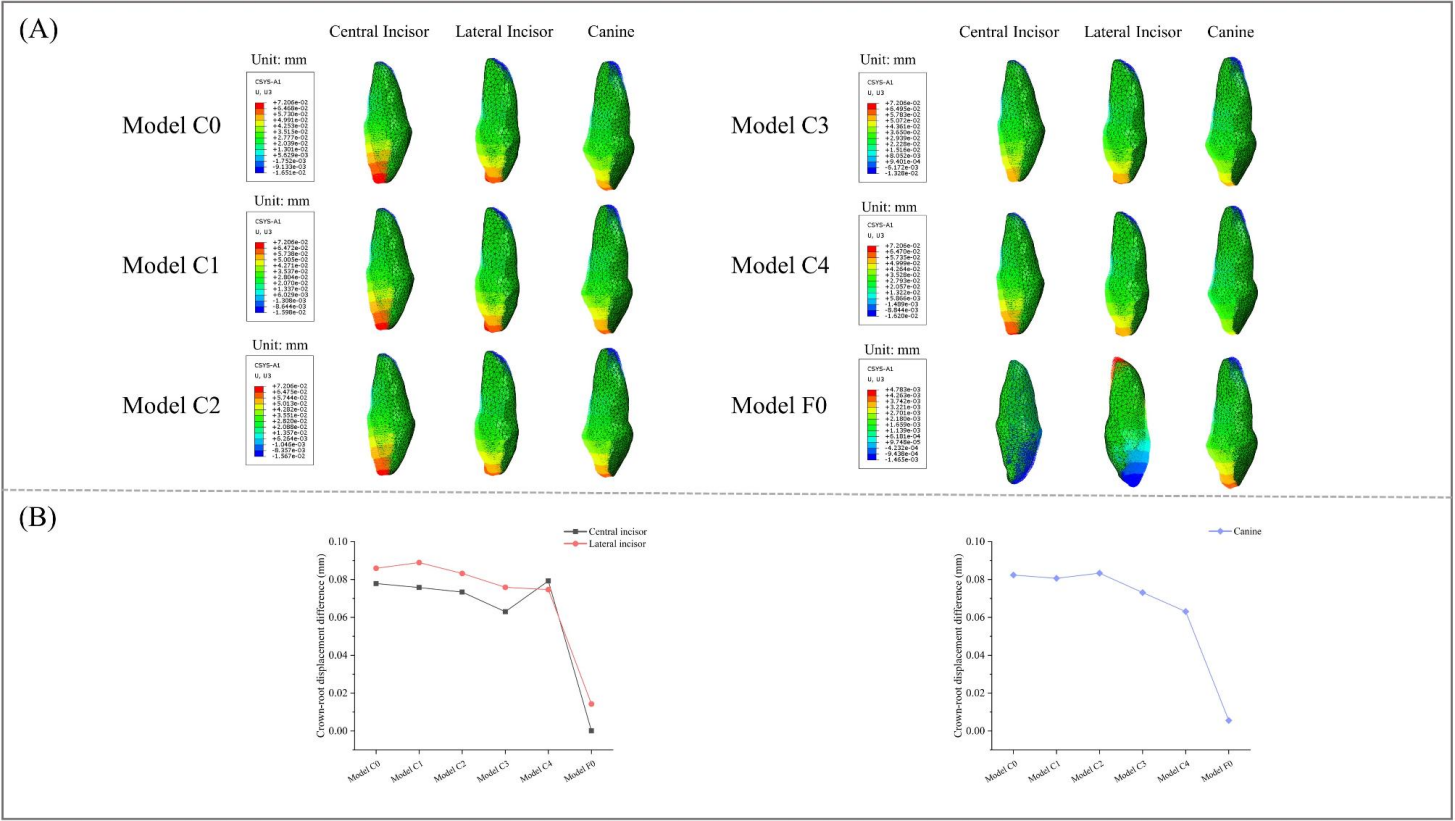
**A**) Displacement tendencies of central incisor, lateral incisor, and canine in sagittal dimension. (**B**) Crown-root displacement difference of central incisor, lateral incisor, and canine in sagittal dimension

**Fig. 5:**
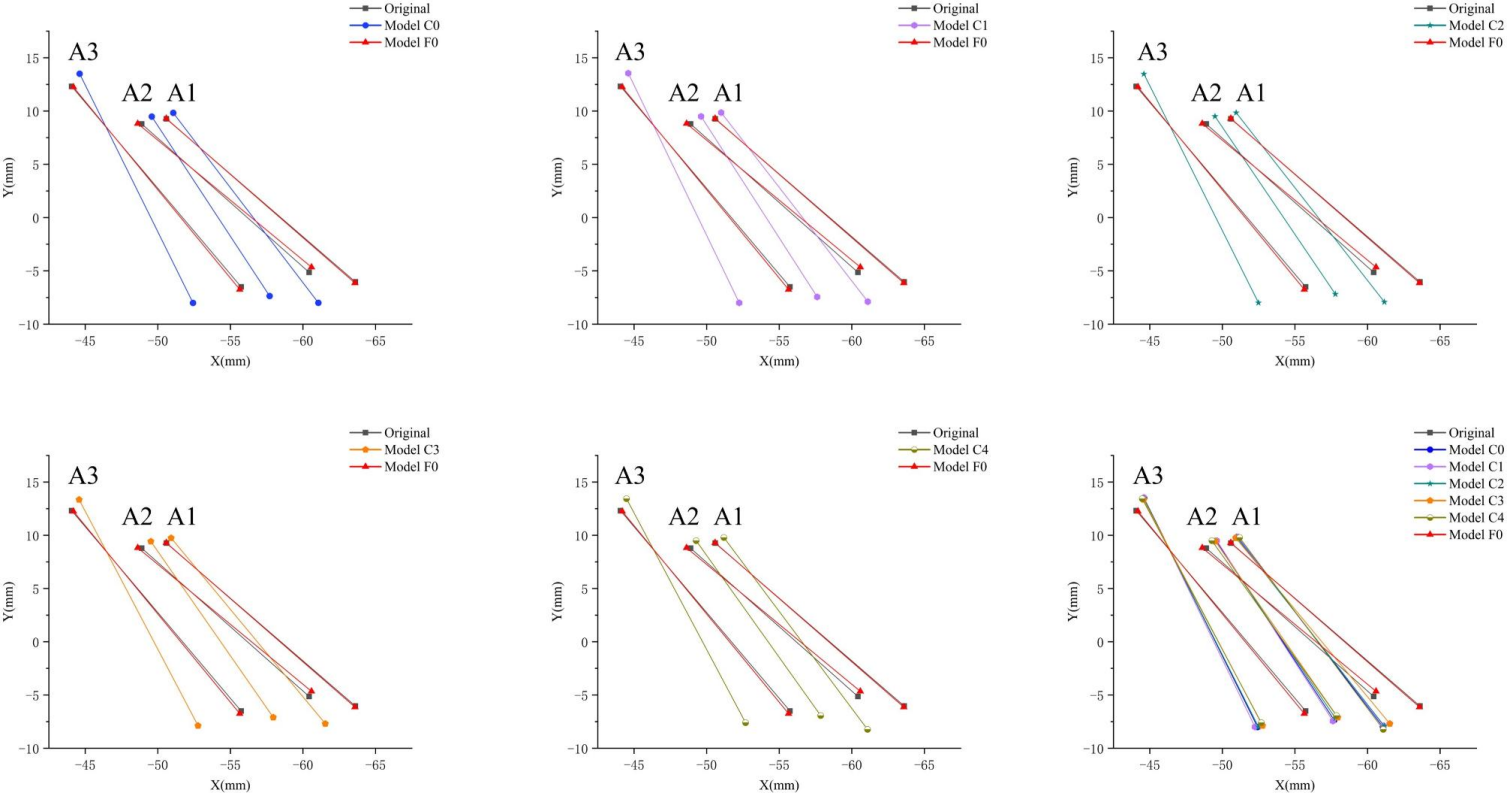
Retraction of central incisor, lateral incisor, and canine in sagittal dimension with different Models. The original place represents the initial position of the anterior teeth in the global coordinate system, and low point represents the incisal midpoint of the anterior and high point represents the root tip of the anterior

The sagittal movement patterns of the central incisor, lateral incisor, and canine were found to be similar under the loading conditions of all five clear aligner models, as depicted in Fig. 4. Notably, these movements exhibited a consistent inclination of the crown towards the lingual side and the root towards the labial side. However, in the fixed appliance model, both the crown and root of the central incisor exhibited buccal movement. The crown of the lateral incisor had buccal movement, while the root moved lingually. Additionally, the canines displayed an opposite trend to that of the lateral incisor. Furthermore, it is worth noting that tooth displacement was significantly lower in the fixed appliance model compared to clear aligner models. Table 2 demonstrates that Model C3 had the smallest crown-root displacement difference for the central incisor at 6.30E-02 mm. For Model C4, the smallest differences were observed for both lateral incisors and canines at 7.47E-02 mm and 6.31E-02 mm respectively. In contrast, in the fixed appliance model, these differences were measured at 1.34E-04 mm for central incisors, 1.43E-02 mm for lateral incisors, and 5.55E-03 mm for canines respectively (Fig. 4). The sagittal retraction of central incisors, lateral incisors and canines under different retraction models was visually demonstrated through a series of figures depicting their initial positions as well as post-retraction positions using both clear aligners and fixed appliances (Fig. 5). For a better understanding of the displacement of the teeth, these movements were magnified 50 times.

**Table 2:** Displacement of crown and root of the maxillary anterior teeth under different loading models in sagittal direction (mm)

		Central incisor			Lateral incisor			Canine	
	Crown	Root	Difference	Crown	Root	Difference	Crown	Root	Difference
Model C0	6.39E-02	-1.40E-02	7.79E-02	6.71E-02	-1.88E-02	8.59E-02	6.13E-02	-2.11E-02	8.24E-02
Model C1	6.22E-02	-1.36E-02	7.57E-02	6.94E-02	-1.96E-02	8.90E-02	6.04E-02	-2.02E-02	8.07E-02
Model C2	6.08E-02	-1.25E-02	7.33E-02	6.51E-02	-1.81E-02	8.32E-02	6.21E-02	-2.12E-02	8.33E-02
Model C3	5.19E-02	-1.11E-02	6.30E-02	5.91E-02	-1.67E-02	7.59E-02	5.45E-02	-1.87E-02	7.31E-02
Model C4	6.44E-02	-1.54E-02	7.98E-02	5.86E-02	-1.50E-02	7.36E-02	4.50E-02	-1.59E-02	6.09E-02
Model F0	-1.12E-04	-2.46E-04	1.34E-04	-1.01E-02	4.22E-03	1.43E-02	4.09E-03	-1.46E-03	5.55E-03

### Comparison of the maximum displacements of the central incisor, lateral incisor, and canine in vertical dimension

**Fig. 6:**
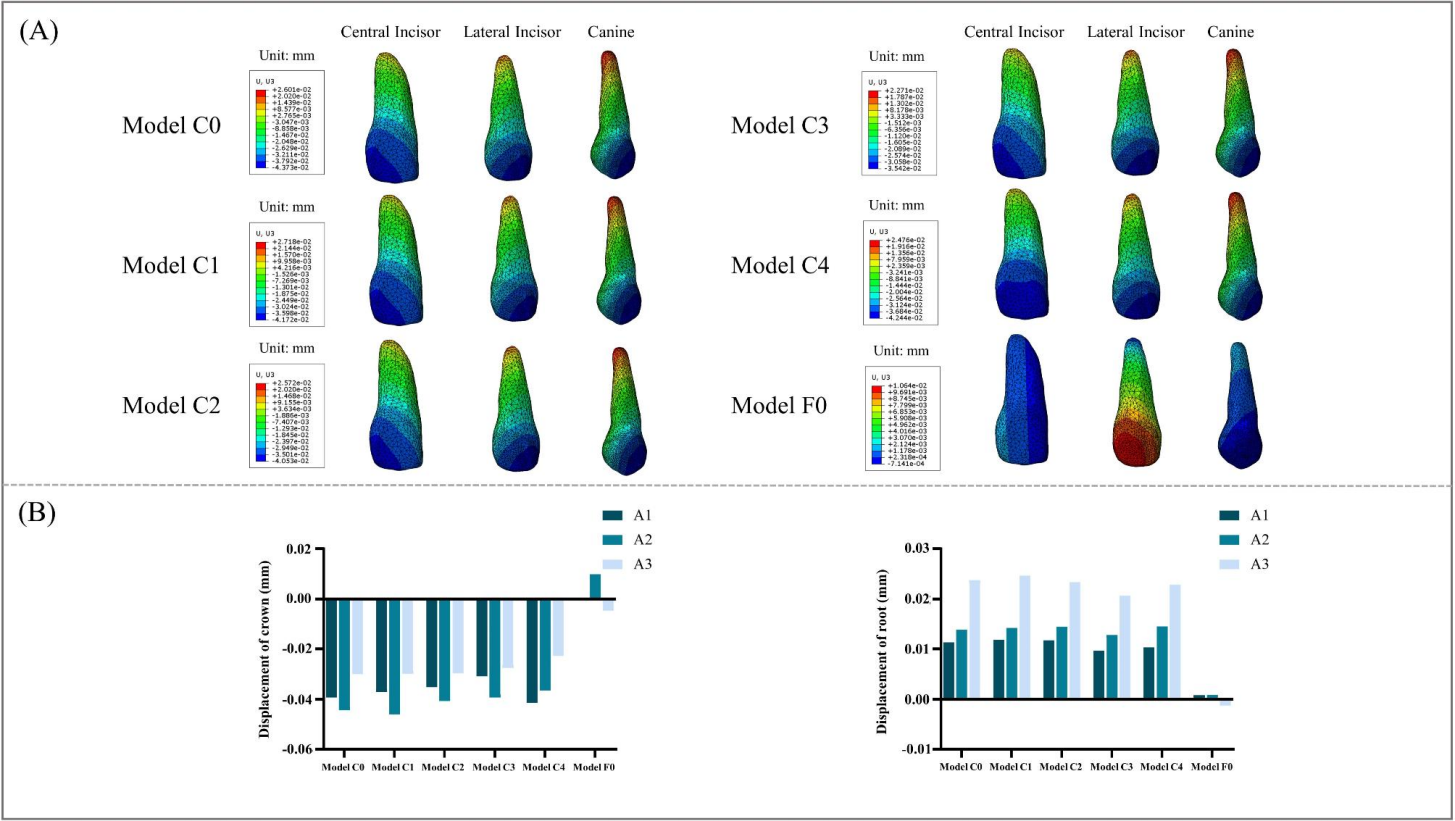
(**A**) Displacement tendencies of central incisor, lateral incisor, and canine in vertical dimension. (**B**) Crown and root displacement of central incisor, lateral incisor, and canine in vertical dimension

As depicted in Fig. 6, displacement tendencies were compared for the central incisor, lateral incisor, and canine in terms of crown and root displacement along the vertical dimension. In Table 3, among the clear aligner models, Model C3 exhibited the smallest crown displacement for the central incisor (-3.08E-02 mm). Similarly, Model C4 showed the smallest displacements for both lateral incisor (-3.65E-02 mm) and canine (-2.27E-02 mm). In contrast, within the fixed appliance model, crown displacements were measured as 5.26E-04 mm for central incisors, 9.77E-03 mm for lateral incisors, and − 4.69E-03 mm for canines. Vertically speaking, all five clear aligner models demonstrated a tendency towards extrusion of anterior teeth; whereas in the fixed appliance model, there was an inclination towards intrusion of central and lateral incisors alongside extrusion of canines.

**Table 3:** Displacement of crown and root of the maxillary anterior teeth under different loading models in vertical direction (mm)

		Central incisor		Lateral incisor		Canine
	Crown	Root	Crown	Root	Crown	Root
Model C0	-3.93E-02	1.13E-02	-4.44E-02	1.38E-02	-3.00E-02	2.37E-02
Model C1	-3.71E-02	1.19E-02	-4.61E-02	1.42E-02	-2.98E-02	2.47E-02
Model C2	-3.53E-02	1.17E-02	-4.07E-02	1.44E-02	-2.96E-02	2.33E-02
Model C3	-3.08E-02	9.66E-03	-3.92E-02	1.28E-02	-2.75E-02	2.06E-02
Model C4	-4.14E-02	1.03E-02	-3.65E-02	1.45E-02	-2.27E-02	2.28E-02
Model F0	5.26E-04	7.59E-04	9.77E-03	8.37E-04	-4.69E-03	-1.29E-03

### Comparison of the maximum displacements of the second premolar, first molar, and second molar in sagittal and vertical dimension

**Fig. 7:**
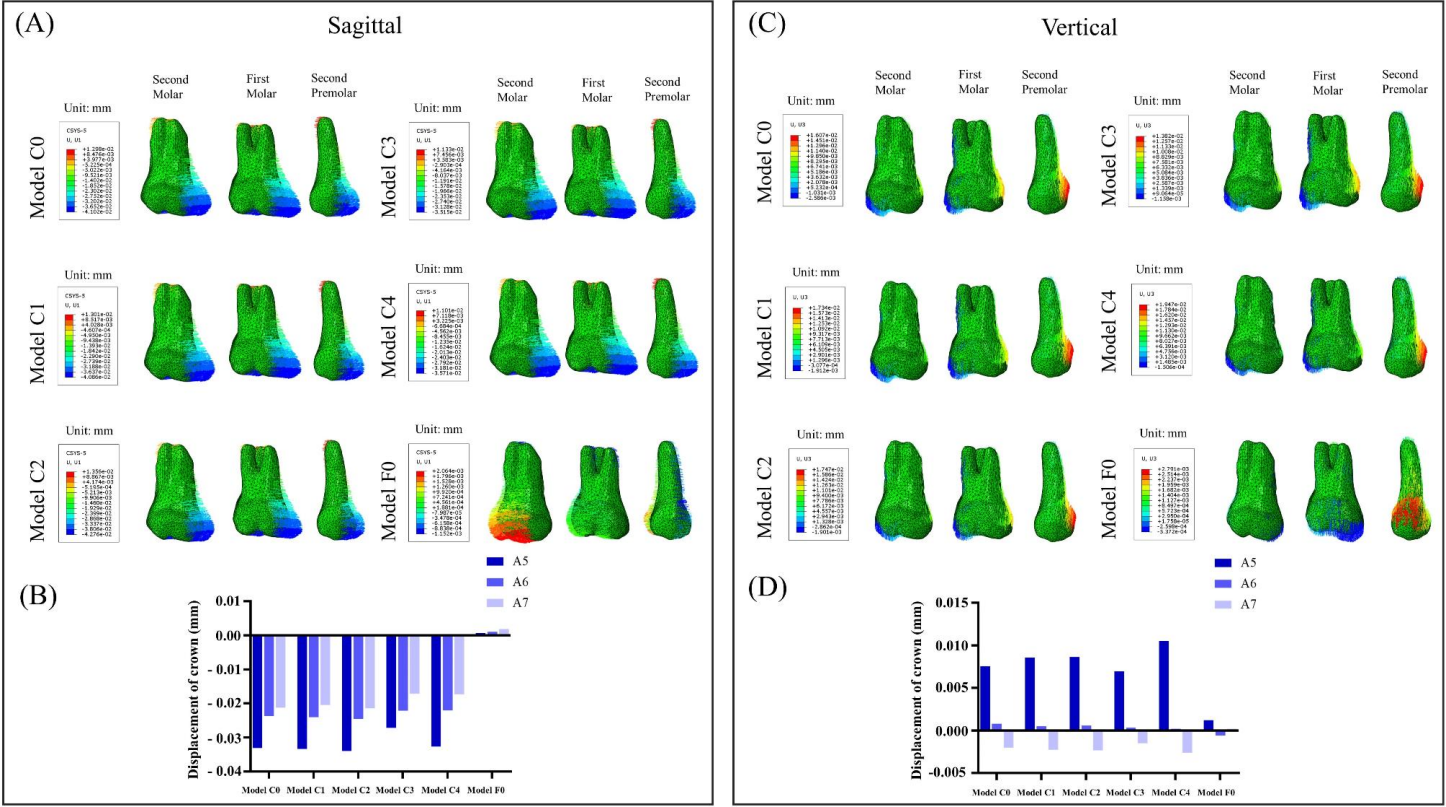
(**A**) Displacement tendencies of second premolar, first molar, second molar in sagittal dimension. (**B**) Crown displacement of second premolar, first molar, second molar in sagittal dimension. (**C**) Displacement tendencies of second premolar, first molar, second molar in vertical dimension. (**D**) Crown displacement of second premolar, first molar, second molar in vertical dimension

As shown in Fig. 7, sagittally, the movement trend of the posterior teeth was similar in the five clear aligner models, all showed an inclined movement trend of the crown toward the mesial and the root toward the distal. In the fixed appliance model, the crown of the posterior teeth showed a tendency to move distally in the sagittal direction (Fig. 7, A). As shown in Table 4, in the clear aligner models, the smallest displacement of the crown of second premolar and second molar in sagittal dimension were observed in Model C3, which were − 2.72E-02 mm and − 1.72E-02 mm. The smallest displacement of the crown of first molar was observed in Model C4, and were − 2.21E-02 mm. The displacement of the crown of second premolar, first molar and second molar in the fixed appliance model were 7.24E-04 mm, 1.05E-03 mm, and 1.78E-03 mm, respectively. Vertically, the movement trend of the posterior was similar in the clear aligner models. The second premolar showed a tendency to intrude, and the first molar was intrusive except for Model C4. The second molar had a tendency to extrude. In the fixed appliance model, the second premolar showed a tendency to intrude, while the first molar and second molar showed a tendency to extrude (Fig. 7, C). In the clear aligner models, the smallest displacement of the crown of second premolar and second molar in vertical dimension were observed in Model C3, which were 6.98E-03 mm -3.08E-02 mm and − 1.50E-03 mm. The displacement of the crown of second premolar, first molar and second molar in the fixed appliance model were 1.23E-03 mm, -5.82E-04 mm, and − 6.25E-05 mm, respectively (Table 5).

**Table 4:** Displacement of crown of the maxillary posterior teeth under different loading models in sagittal direction (mm)

	Second premolar	First molar	Second molar
	Crown	Crown	Crown
Model C0	-3.31E-02	-2.37E-02	-2.13E-02
Model C1	-3.34E-02	-2.41E-02	-2.05E-02
Model C2	-3.40E-02	-2.46E-02	-2.15E-02
Model C3	-2.72E-02	-2.22E-02	-1.72E-02
Model C4	-3.27E-02	-2.21E-02	-1.73E-02
Model F0	7.24E-04	1.05E-03	1.78E-03

**Table 5:** Displacement of crown of the maxillary posterior teeth under different loading models in vertical direction (mm)

	Second premolar	First molar	Second molar
	Crown	Crown	Crown
Model C0	7.57E-03	8.19E-04	-2.01E-03
Model C1	8.57E-03	5.35E-04	-2.28E-03
Model C2	8.64E-03	5.89E-04	-2.31E-03
Model C3	6.98E-03	3.58E-04	-1.50E-03
Model C4	1.05E-02	-5.49E-05	-2.61E-03
Model F0	1.23E-03	-5.82E-04	-6.25E-05

### Comparison of the maximum displacements and von mises stress in the clear aligners and fixed appliance

**Fig. 8:**
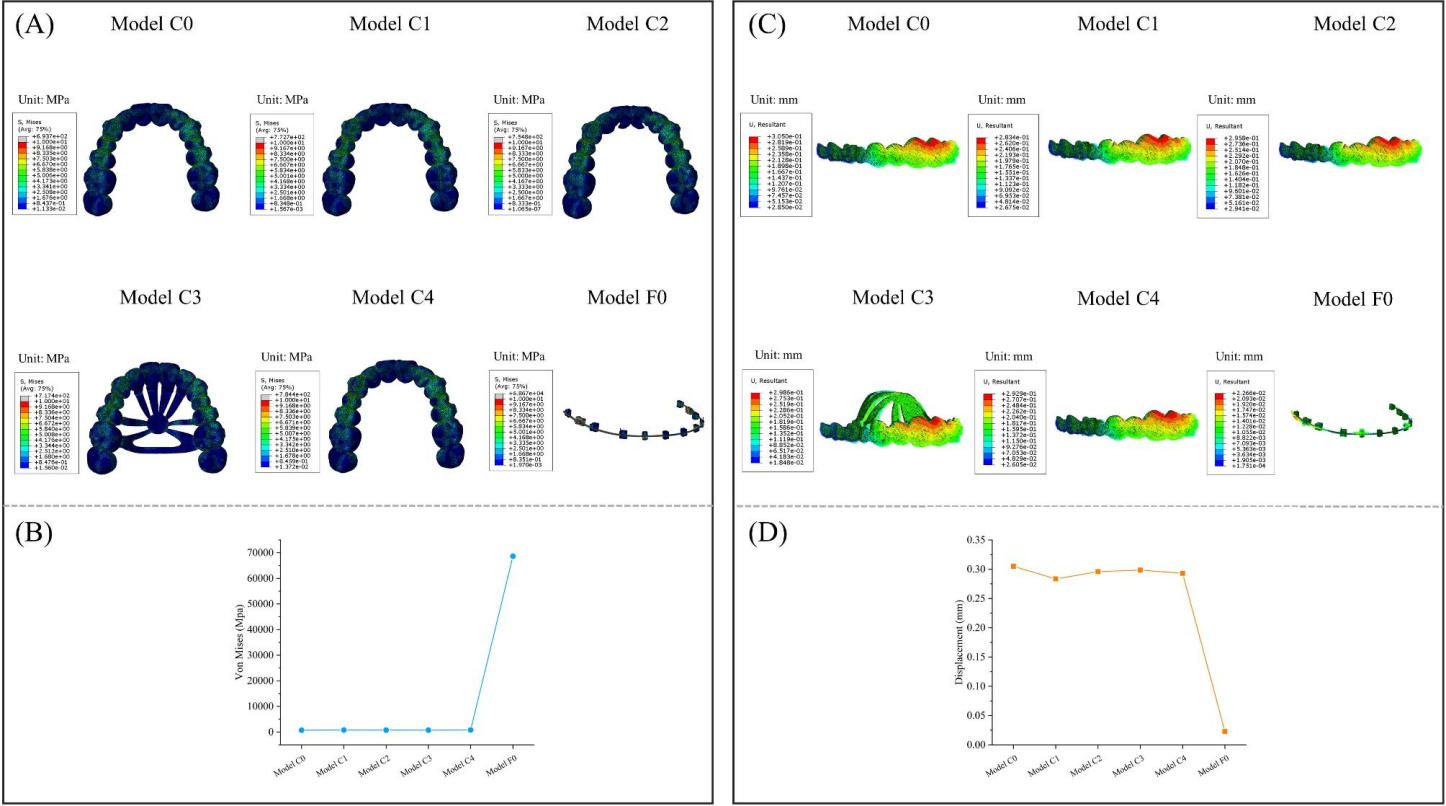
(**A**) The von Mises distribution (blue to gray reflects lower to higher stress) of clear aligners and fixed appliance. (**B**) Stress value for maximum von Mises of the clear aligners and fixed appliance. (**C**) Displacement tendencies of clear aligners and fixed appliance. (**D**) The maximum displacement of clear aligner and fixed appliance

The maximum von mises of the clear aligner was 693.733 MPa, 772.713 MPa, 754.77 MPa, 717.365 MPa, and 784.445 MPa, respectively. The maximum von mises of the fixed appliance was 68668.1 MPa (Fig. 8, B). The stress distribution in the clear aligner model was similar, with stresses concentrated at the aligners corresponding to the canine, first premolar, and second premolar teeth, especially at the teeth adjacencies. The stress of the fixed appliance was located primarily on the archwire and the brackets corresponding to the first molar. (Fig. 8, A). The maximum displacement of the five clear aligner models was 0.304961 mm, 0.283423 mm, 0.295801 mm, 0.298634 mm, 0.292909 mm, respectively. The maximum displacement of the fixed appliance was 0.022658 mm. The displacement trends of the clear aligners in the clear aligner models were similar, with a tendency to move buccally and toward the occlusal direction. In the fixed appliance model, there was a tendency for the corresponding position of the lateral incisors of the archwire to be deformed towards the root and lingual side. Moreover, the corresponding position of the second premolar of the arch wire had a trend of buccal dislocation. Since the archwire corresponding to the position of the first molar was constrained by the buccal canal, the deformation and extrusion were obvious under the retraction force.

### Comparison of von mises stress in the PDL of the central incisor, lateral incisor, canine, second premolar, first molar, and second molar

**Fig. 9:**
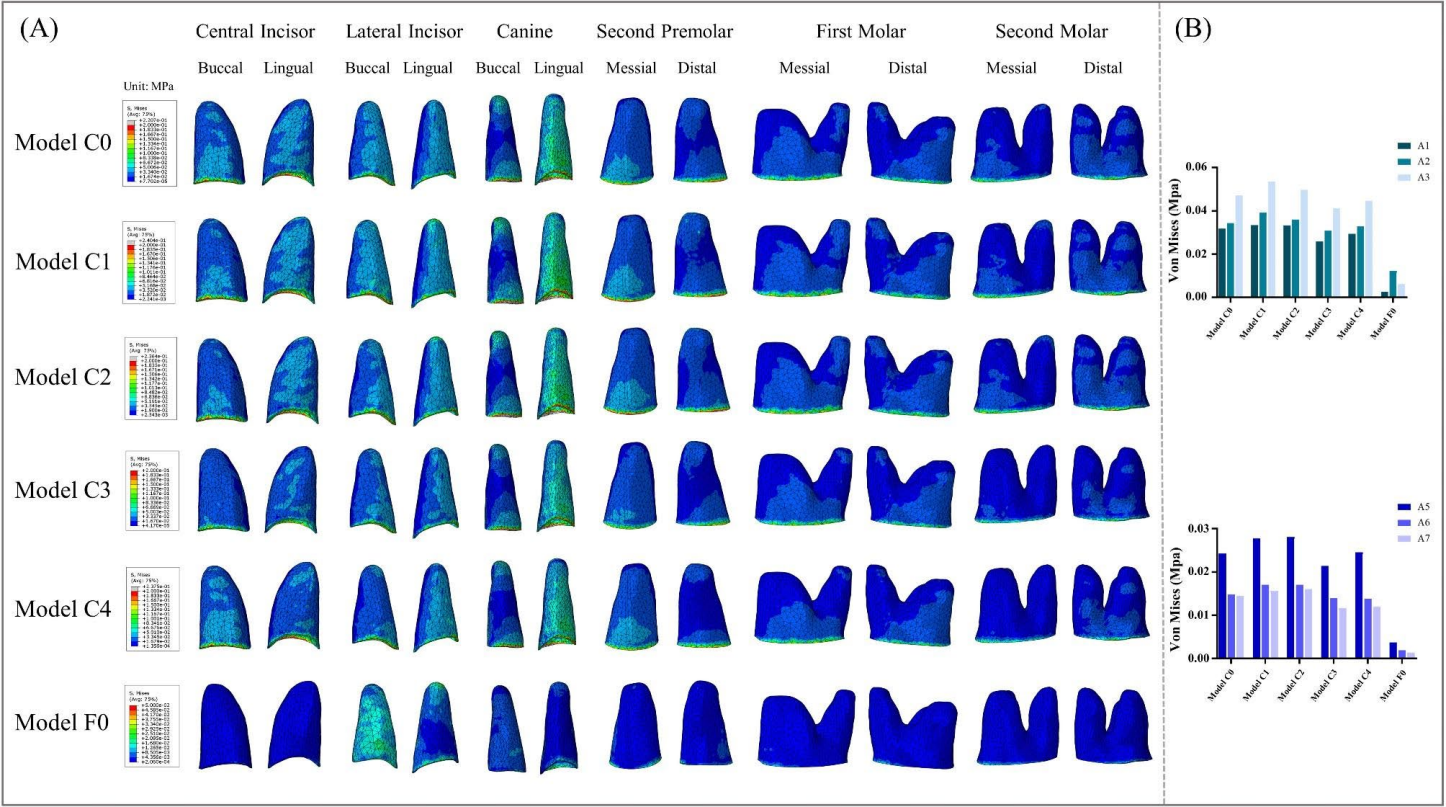
(**A**) Distribution of von Mises stresses in the PDL of the central incisor, lateral incisor, canine, second premolar, first molar and second molar. (**B**) Stress value for average von Mises in the PDL of the central incisor, lateral incisor, canine, second premolar, first molar and second molar

As depicted in Fig. 9, the average von mises and stress distribution of PDL in six retraction models were compared. The stress magnitude and stress distribution on PDL were similar in the five clear aligner models. Among the five clear aligner models, the lowest stress of the PDL of the central incisor, lateral incisor, canine, second premolar and second molar appeared in Model C3, which were 0.025871 MPa, 0.030915 MPa, 0.041213 MPa, 0.021395 MPa and 0.011692 MPa, respectively. Model C4 had the lowest PDL stress in the first molar, which was 0.013860 MPa. The stress on the PDL of the central incisor, lateral incisor, canine, second premolar, first molar, and second molar in the fixed appliance retraction model were 0.00256 MPa, 0.012276 MPa, 0.006295 MPa, 0.003738 MPa, 0.001902 MPa, and 0.001394 MPa, respectively. The PDL stress distribution was obviously different between the clear aligner model and the fixed appliance model. In the clear aligner models, the stress was mainly located in the anterior teeth and the second premolar, and the PDL stress of the first molar and the second molar decreased significantly. In the fixed appliance model, the stress was mainly concentrated on the lateral incisor, canine and second premolar. In the clear aligner models, the stress on the PDL of the central incisors, lateral incisors and canines was located on the buccolingual side and concentrated mainly in the cervical position. The stress on the PDL of the second premolar was mainly distributed in the cervical of the mesial and distal of the root surface. The stress of PDL on the first and second molars was concentrated in the cervical region of the mesial and distal of the root surface. In the fixed appliance model, the stress of PDL of the lateral incisor was mainly located on the buccal side, with a concentration in the apical and lingual cervical locations. For canine, the PDL stress was mainly located on the buccal side and distributed more uniformly. The PDL stress of the second premolar was mainly concentrated on the buccolingual cervical region.

## Discussion

In this study, we conducted numerical simulations to investigate the process of anterior retraction in different orthodontic designs and compared the biomechanical differences among various invisible orthodontic devices during anterior retraction. Additionally, we compared the clear aligner retraction model with the fixed appliance retraction model. The results showed minimal biomechanical disparities among different clear aligner models. The additional force systems did not alter the trend of tooth movement in clear aligner models but rather adjusted both anterior and posterior teeth displacement during retraction. Model C3 demonstrated superior torque control and provided enhanced protection for posterior anchorage teeth compared to other four clear aligners. The clear aligner and fixed appliance exhibited distinct biomechanical properties, with the latter showing superior anterior torque control and posterior anchorage tooth protection compared to the former.

The clear aligner models consistently demonstrated lingual tipping and extrusion in the anterior teeth, as well as a similar movement pattern in the posterior teeth with their crowns tilting towards the mesial side, consistent with the findings reported by Wang et al. [50, 51]. Retraction of the anterior teeth using clear aligners leads to a roller-coaster effect of tooth movement [5, 50, 52, 53]. The additional force systems in the study did not change the observed trend of tooth movement in the model, but they did introduce some variation in the displacement magnitude of both anterior and posterior teeth. In Liu et al.‘s study, the utilization of anterior mini-screws and elastics demonstrated their efficacy in achieving incisor intrusion and palatal root torquing [7]. Consistent with their findings, our experimental group Model C2 also exhibited superior control over the anterior teeth in terms of torque and vertical control when compared to Models C0 and C1. However, the observed trend was not as pronounced, potentially due to variations in force magnitude and application method. Liu’s study revealed that longer anterior teeth experienced less tipping [53], which aligns with the results obtained from our control group Model C0. Furthermore, our experimental group Model C3 deviated from this trend by showcasing a smaller displacement tendency for central incisors with shorter roots compared to canines. Additionally, all anterior teeth displayed a decreasing sagittal tipping displacement trend. The results indicate that Model C3 exhibited the most precise torque and vertical control for central incisors, as evidenced by its minimal crown-root displacement difference and vertical displacement. This phenomenon can be attributed to the stabilizing and cushioning effect of the palatal plate structure during the retraction process. The displacement of the posterior teeth in the sagittal and vertical directions was effectively minimized, indicating optimal protection for posterior retention. This was related to the role of the palatal plate in combining with the posterior teeth to form a stronger anchorage unit. The Model C4 had the best torque control and vertical control for lateral incisor and canine, which was due to the role of the lingual retractor.

The initial displacement tendency of teeth in the fixed appliance model was significantly different from that in the clear aligners. The fixed appliance had the most pronounced effect on the lateral incisor, causing a labial tipping with intrusion of the lateral incisor. The reason for this was the proximity of the traction point to the lateral incisors and the fact that the lateral incisors exhibited a relatively smaller periodontium compared to other anterior teeth in general condition [54, 55]. Moreover, the posterior teeth showed a tendency to move distally, due to the backward frictional force exerted by the archwire on the posterior teeth when closing the gap. On the other hand, the displacement magnitude of the teeth in the fixed appliance model was significantly less than in the clear aligner models. This was consistent with previous studies that clear aligner was not as good as fixed appliance in controlling tooth torque and posterior anchorage protection [56, 57]. We explored the reasons for this by comparing the stress and displacement of clear aligners with fixed appliance. From Fig. 7, A. it can be seen that the clear aligners had greater stress at the joint of adjacent teeth and a tendency to fall off in the occlusion direction, which was in agreement with the findings of Meng et al. [29]. However, the maximum von mises stress of clear aligner was still significantly less than that of fixed appliance. When fixed appliances were subjected to forces, most of the forces were carried by the fix appliances themselves, so the forces transmitted to the teeth were significantly reduced. However, when clear aligners were deformed, the force acted directly on the tooth surface and there was no force decay process. Moreover, Fig. 7, B showed that the deformation of the clear aligners was significantly greater than that of the fixed appliance, about fifteen times greater. The greater the deformation of the clear aligner the greater the force applied to the tooth. In agreement with Danilee K. B et al., clear aligner was not stiff enough to maintain the tipping tendency compared to fixed appliance, which can lead to a significant roller-coaster effect [58]. The clear aligner approach and the fixed appliance approach still exhibit a disparity; nevertheless, this study offered a developmental direction and established a theoretical foundation for future non-invasive, aesthetically pleasing, comfortable, and efficient modalities of clear aligner treatment. Improvements in materials, design refinements, and 3D printing technology have made it possible to create clear aligner with better orthodontic capabilities by improving design parameters such as aligner configuration, strength, elasticity, or thickness [16, 17, 59].

Root absorption can result from excessive stress concentration, and it has been reported that 91% of teeth underwent some degrees of root resorption after orthodontic treatment [60]. Stress distribution of PDL was consistent with the trend of tooth movement [30]. Since the five clear aligner models had the same trend of movement, the stress distribution in PDL was also roughly the same. For the clear aligner models, the stress of the central incisors, lateral incisors and canines was mainly concentrated on the cervical of the buccal and lingual root surfaces and apical regions, which was consistent with the findings of Liu [7]. In addition, the stress of the second premolar, first molar and second molar was mainly concentrated on the cervical of the mesial and distal root surfaces. The root surfaces of central and lateral incisors are smaller than those of premolars and molars, making them more susceptible to root resorption [45]. In Model C3, the PDL stress of anterior teeth was smaller than that in the other clear aligner models, and the stress distribution area was also smaller. The results suggested that the modified palatal plate clear aligner helped reduce the risk of root resorption during anterior retraction. In the fixed appliance model, the lateral incisor was subjected to the greatest stress, and the stress mainly concentrated on the buccal surface, the root tip and the cervical of the lingual surface. However, the stress was still smaller than that in the clear aligner models. Consistent with Tang et al., the stress of the PDL in the fixed appliance model was significantly less than that in the clear aligner models [61]. Accordingly, this may be an obvious risk factor for root resorption caused by clear aligner therapy.

However, it is imperative for this study to acknowledge its potential limitations. The limitations of this simulated study remain, as it can only explain the initial effects of stress distribution and displacement patterns on teeth when analyzing orthodontic appliance force systems. Simplification and assumption pose evident limitations in the context of finite element analysis. Frequently, more intricate anatomical structures are disregarded during the modeling phase. Another concern arises when attempting to accurately represent not only the anatomy but also the morphology of tested tissues, where simplifications are commonly employed [62]. As digital simulation technology advances, our next endeavor is to achieve a more precise and comprehensive simulation of the orthodontic process. Additionally, replicating exactly the same living substance in a mechanical model proves virtually impossible; hence further investigation into finite element analysis through extensive clinical studies is necessary to quantitatively validate our findings. Moreover, combining FE analysis with clinical studies for mutual validation will enhance the significance of this study, which represents our subsequent step. The modified palatal plate clear aligner we designed is too monolithic, but this study provides direction for future research. Moreover, we will further improve the configuration, strength, elasticity, thickness and other design parameters of the clear aligner to explore the modified clear aligner with better efficacy.

## Conclusions

After conducting preliminary research, we have arrived at the following conclusions:

1. The teeth movement pattern remained consistent across all five clear aligners, characterized by lingual tipping and extrusion of anterior teeth, as well as mesial tipping of posterior teeth during anterior retraction.
2. Fixed appliances exhibit superior control over torque in anterior teeth and provide better protection against anchorage loss in posterior teeth compared to invisible appliances.
3. The implementation of an additional force system in clear aligners did not alter the observed trend of tooth movement, but it did exert an influence on the magnitude of tooth displacement. Specifically, modified palatal plate structure clear aligner Model C3 demonstrated enhanced torsional control and improved preservation of posterior dental anchorage.

### Electronic supplementary material

Below is the link to the electronic supplementary material.

**Supplementary Material 1: Additional file 1:** Supplementary Figure 1. Different views of the Model C4: (A) Occlusal view of Model C4, red arrow represents the applied force loading (150 g) from hook to the 3D printed palatal plate. (B) Palatal view of Model C4. (C) Occlusal view of the 3D printed lingual retraction hook and the 3D printed palatal plate. (D) Sagittal view of Model C4, the distance from the traction point to the occlusion plane is 18.54 mm. **Additional file 2:** Supplementary file 1. Determination of the center of resistance (CR) and Determination of the height of lingual retraction hook. **Additional file 3:** Supplementary Figure 2. (A) Displacement tendencies of central incisor, lateral incisor, and canine in sagittal dimension. (B) Crown-root displacement difference of central incisor, lateral incisor, and canine in sagittal dimension

## Data Availability

The datasets used and/or analysed during the current study available from the corresponding author on reasonable request.
