# Supplementary material for: Comparative assessment of orthodontic clear aligner versus fixed appliance for anterior retraction: a finite element study
Source: BMC Oral Health. 2024 Jan 13;24:80. doi: 10.1186/s12903-023-03704-6 (PMC10787995; doi:10.1186/s12903-023-03704-6)

**Supplementary Information**

**Additional file 1: Supplementary Figure 1.**

**Additional file 2: Supplementary file 1.**

**Additional file 3: Supplementary Figure 2.**

**Supplementary Figure 1.** Different views of the Model C4.


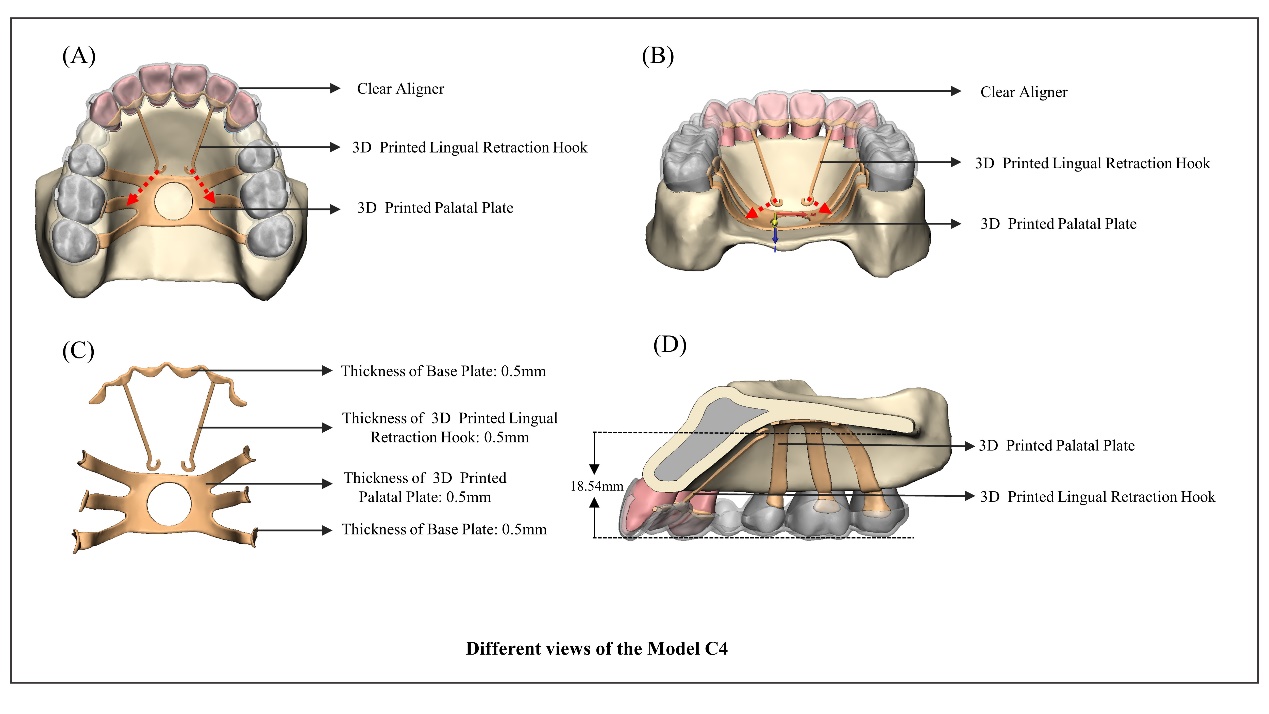


**Additional file 2: Supplementary file 1.** Determination of the center of resistance (CR) and Determination of the height of lingual retraction hook.

**Determination of the center of resistance (CR)**

In this study, the height of the lingual retractor was determined by initially establishing the vertically oriented height of the center of resistance (CR) within the retraction unit. The retraction unit models were assigned with the property of rigidity. The mesial-distal truncated surfaces of the maxilla were firmly constrained (Fig 2, A). To determine the vertical height of the center of resistance (CR) of the retraction unit, a 100 g horizontal force was applied in the near median sagittal plane, parallel to the occlusal plane, for lingual retraction (Fig 2, A). Furthermore, the point of force application (level 0) was positioned on the alveolar ridge roof of the posterior teeth at a distance of 7.69 mm from the incisal edge (Fig 2, B). Starting from level 0 and moving towards the root at intervals of 1mm perpendicular to the occlusal plane up to level 7, which approached close to the root apex of anterior tooth. All components were imported into finite element (FE) software for calculations. The difference between the displacement of the root tip and crown displacement was defined as the crown-root differential displacement. The center of resistance (CR) level is defined as the point where the differential displacements of anchorage units are close to 0. After step-by-step subdivision of the loading calculation, we determined that the vertical position of the center of resistance (CR) is at 4.85mm.

**Determination of the height of lingual retraction hook**

Our study revealed that the combined utilization of a lingual retraction hook and clear aligner failed to achieve comprehensive movement through the center of resistance (CR), as the retraction force exerted by the clear aligner resulted in an undesired displacement towards the root direction. Consequently, we conducted additional force calculations at incremental distances of 1mm, 2mm, 3mm, 4mm, 5mm, and 6mm above the CR positions. The maximum position achieved was 6 mm above the CR due to the proximity of this position to the patient's hard palate, which limited further extension of the retraction hook. It was observed that there was a similar pattern of movement in the anterior teeth from the level of the CR to 6 mm above it, and a gradual decrease in crown-root displacement difference among these teeth (Supplementary figure 2). Ultimately, for achieving a more comprehensive movement of the anterior teeth, we selected a position 6 mm above the CR (approximately 18.54mm above occlusal plane) as the length for lingual retraction hook (Fig 3).

**Additional file 3: Supplementary Figure 2.** (A) Displacement tendencies of central incisor, lateral incisor, and canine in sagittal dimension. (B) Crown-root displacement difference of central incisor, lateral incisor, and canine in sagittal dimension.


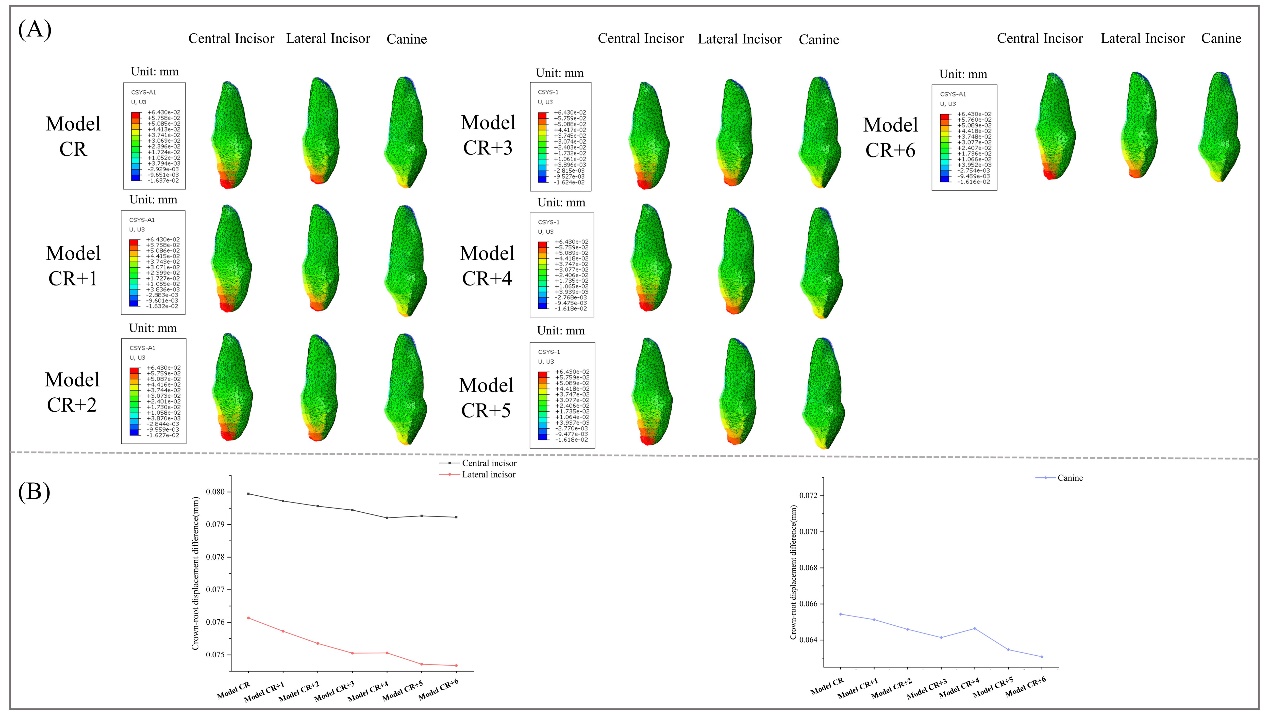

Supplement: Supplementary file 1 — Supplementary Material 1: Additional file 1: Supplementary Figure 1. Different views of the Model C4: (A) Occlusal view of Model C4, red arrow represents the applied force loading (150 g) from hook to the 3D printed palatal plate. (B) Palatal view of Model C4. (C) Occlusal view of the 3D printed lingual retraction hook and the 3D printed palatal plate. (D) Sagittal view of Model C4, the distance from the traction point to the occlusion plane is 18.54 mm. Additional file 2: Supplementary file 1. Determination of the center of resistance (CR) and Determination of the height of lingual retraction hook. Additional file 3: Supplementary Figure 2. (A) Displacement tendencies of central incisor, lateral incisor, and canine in sagittal dimension. (B) Crown-root displacement difference of central incisor, lateral incisor, and canine in sagittal dimension [file 12903_2023_3704_MOESM1_ESM.docx]
